# Changes in anxiety and depression symptoms during the Covid-19 lockdown in the Netherlands. The moderating role of pre-existing mental health, employment situation and alcohol consumption

**DOI:** 10.1007/s00127-023-02480-6

**Published:** 2023-04-06

**Authors:** Lluís Mangot-Sala, Nynke Smidt, Aart C. Liefbroer

**Affiliations:** 1 Netherlands Interdisciplinary Demographic Institute (NIDI)–Royal Netherlands Academy of Sciences (KNAW), Lange Houtstraat 19, 2511 CV The Hague, The Netherlands; 2 Department of Epidemiology, University Medical Center Groningen (UMCG), University of Groningen (RUG), Groningen, The Netherlands; 3 Department of Sociology, Vrije Universiteit Amsterdam (VU), Amsterdam, The Netherlands

**Keywords:** Coronavirus, Mental health, Pandemic, Work, Heavy drinking, Alcohol abuse

## Abstract

**Purpose:**

Evidence suggests an increase of depression and anxiety symptoms during the Covid-19 pandemic but most studies relied on cross-sectional designs and/or small samples, and they often overlooked subgroup effects in the impact of the lockdown. We investigated the effect of the pandemic on depression and anxiety symptoms, and whether it differed by employment situation and alcohol consumption.

**Methods:**

This longitudinal study used 23 waves of the Covid-Questionnaire (April 2020—July 2021), within the Lifelines cohort from the Netherlands (*n* = 76,254). Depression and anxiety symptoms were combined in a “mental health score”. Linear fixed-effects models were fitted to analyse trends in mental health throughout the observation period. The moderating role of pre-existing mental health, employment situation, and alcohol consumption was tested.

**Results:**

Depression and anxiety symptoms fluctuated considerably during the observation period, with clear peaks in winter 2021, during the strictest lockdown period. Moreover, temporal patterns differed by employment situation and alcohol consumption patterns, suggesting that various subgroups reacted to the pandemic and the lockdown in different ways.

**Conclusion:**

Lockdowns increased depression and anxiety symptoms in the Netherlands. The effect was particularly strong for unemployed individuals, those with risky alcohol consumption patterns and those with pre-existing mental health disorders.

**Supplementary Information:**

The online version contains supplementary material available at 10.1007/s00127-023-02480-6.

## Introduction

Two years of the Covid-19 pandemic are likely to have affected mental health. Lockdowns -i.e. preventive measures implemented to control the spread of the virus- led to reduced social interactions and higher levels of social isolation [1, 2]. Moreover, they meant spending long hours at home for many people, which may also have had mental health consequences. Systematic reviews have consistently shown that the prevalence of mental health problems increased during lockdown periods [3, 4].

However, not all results point in the same direction. Some studies showed no evidence of a negative effect of lockdowns on mental health: a longitudinal study covering the first six weeks of the lockdown in the United Kingdom showed that depression and anxiety decreased during that period [5], and another study showed that mental health in Germany improved during the first eight weeks of lockdown [6]. However, these studies covered only a short period of time at the beginning of the lockdown, and cannot be generalized to the whole pandemic period. A British study explained this pattern by suggesting an initial “shock” caused by the first lockdown, which led to more prevalent mental health symptoms, followed by improvement in subsequent weeks [1].

Although mental health disorders are complex, multifactorial phenomena, there is agreement that situations, in which individuals do not perceive real control over their lives, can trigger symptoms of depression or anxiety [7]. This is particularly true for the employment domain [8]. Unemployment has been shown to lead to poor mental health [9], partly because it often entails substantial uncertainty [1, 10, 11]. Therefore, we expect that being laid off in the already uncertain context of a pandemic will lead to a stronger mental health deterioration, as the impact of both unemployment and the pandemic are likely to interact and “accumulate” over time [12].

The pandemic also led to more frequent working from home. There is mixed evidence on the mental-health consequences of doing so. While some studies reported stronger negative effects, particularly for those living alone [1], allegedly due to stronger feelings of loneliness and isolation [13], others reported an overall positive effect of working from home, due to greater perceived control over work and better work-life balance [14]. Although the consequences of working from home are likely to depend on numerous factors -e.g. type of job or household composition [15], such a sudden adjustment is likely to have a negative effect on mental health. Hence, we expect that those who worked full-time from home during the pandemic will report a stronger deterioration of their mental health.

Another group that has been proven vulnerable to the negative impact of lockdowns are people with pre-existing chronic conditions [3]. However, evidence regarding the consequences for their mental health is less clear: although those with pre-existing mental disorders reported a higher prevalence of depression and anxiety symptoms [1], whether the effect of lockdowns was stronger for them remains unclear. In fact, one study showed that those with previous mental disorders may have adjusted slightly better to the lockdown, allegedly because their habits may have become “more in sync with a quarantined society” [16]. Yet, the conservation of resources theory suggests that resilience during a disruptive event, such as the Covid-19 pandemic, requires certain resources, among which a fair health status [17]. Thus, we expect those with pre-existing mental health conditions, to suffer a greater deterioration of their mental health.

In addition, the role of lifestyle factors, such as alcohol consumption, in mental health changes during the lockdown remains unclear. While alcohol consumption is considered a coping mechanism for pre-existing depression and anxiety according to self-medication theory [18], it may also increase the risk of mental disorders [19–21], and mood instability, i.e. sudden and frequent fluctuations of mood. These fluctuations are associated with worse treatment outcomes and increased health service use [22]. Since alcohol abuse may interact with the already negative effects of the lockdown, we expect a stronger deterioration of mental health for those reporting risky alcohol consumption during the strictest lockdown periods [21].

Thus far, most of the evidence about the impact of lockdowns on mental health is based on studies using cross-sectional designs [23, 24] or, at best, short observation periods [2, 5, 14, 25], which results in limited evidence of mental health changes over time [22]. Some studies have analyzed fluctuations in mental health symptoms longitudinally [6, 26] but they have relied on rather small samples and limited observations. Meanwhile, studies with larger samples mainly focused on the prevalence of mental health among different subgroups [1, 27] rather than changes over time, or focused on describing temporal trends but did not delve into potential subgroup effects [28]. This study contributes to the literature by analyzing differences across subgroups in the association between lockdowns and depression and anxiety symptoms. To our knowledge, our study is the first to test whether the employment situation individuals faced during the pandemic, as well as their health-related behaviours -i.e. drinking patterns- and outcomes -pre-existing mental health issues moderated the impact of the lockdowns.

This study uses a large sample and 23 waves of panel data gathered frequently during the Covid-19 pandemic, which allows us to examine the development of depression and anxiety symptoms over a 15-month observation period. We aim to advance the existing longitudinal evidence by testing: (1) whether the lockdown affected depression and anxiety symptoms; (2) whether this effect differed by employment situation, and (3) whether this effect differed by alcohol consumption patterns. Furthermore, this study analyses the main determinants of greater fluctuations in depression and anxiety symptoms during the pandemic.

## Methods

The Lifelines Covid-19 Questionnaire was launched within the Lifelines Cohort Study, a large prospective population-based cohort study and biobank in the three northern provinces of the Netherlands, examining in a three-generation design the biomedical, socio-demographic, behavioural, physical and psychological factors contributing to the health and disease of 167,729 individuals living in the north of the Netherlands -composition and characteristics of the sample have been discussed elsewhere [29].

To assess the effects of the pandemic, attitudes towards Covid-19 regulations, and the health behaviours of the study population, the Lifelines Covid-19 cohort was developed. Participants over 18 years with known email addresses (*n* = 140,145) were asked to complete detailed web-based questionnaires about their physical and mental health, living situation and health behaviours. A total of 76,795 individuals completed at least one wave of the questionnaire (54.8% response rate), with an average of 17 observations per person, from 24 waves in total. The characteristics and representativity of the sample are presented in Mc Intyre et al. [30]. For our study, 23 waves of the Covid-19 questionnaire were used -wave 12 had no information on mental health-, covering the period between April 2020 and July 2021. Our final sample consists of 76,254 individuals (61.3% female; mean age 54.4 years; average follow-up of 146.54 days) and 876,874 observations. Data from the same participants in waves 4 and 5 of the Lifelines Cohort Study were used for comparison.

### Measurements

#### Outcome variables

The Lifelines Covid-19 questionnaire is based on DSM-5 criteria and covers most depression and anxiety symptoms using the Mini International Neuropsychiatric Interview (MINI) [31]. However, due to the frequency of data collection within our longitudinal design, the symptom duration does not follow the gold-standard definition for “major depressive disorder” (“for more than two weeks”) and “generalized anxiety disorder” (“at least 6 months”) [31]. Instead, the questionnaire refers to symptoms since the last observation (“in the last 7 days” or “in the last 14 days”, depending on the observation). Therefore, we label these as “symptom scores” rather than disorders.

#### Depression symptom score

This variable ranges from 0 to 8 symptoms: (1) Depressed mood; (2) diminished interest or pleasure in most or all activities; (3) significant weight or appetite changes; (4) insomnia or hypersomnia; (5) psychomotor retardation; (6) feelings of worthlessness or excessive or inappropriate guilt; (7) diminished ability to think or concentrate; (8) recurrent thoughts of death or suicidal ideation.

#### Anxiety symptom score

This variable ranges from 0 to 6 symptoms: (1) Worrying excessively about daily problems; (2) worries present almost every day; (3) worries are hard to set aside and/or prevent concentration; (4) restlessness or feeling “keyed up or on edge”; (5) muscle tension (6) increased irritability.

In addition, both scores were summed to generate a combined *mental health score*, ranging from 0 to 14.

#### Main independent variable

Lockdown period or “days since first lockdown (15th of March, 2020)” was converted into a categorical variable with categories coinciding with every month of the observation period (category 1 = April 2021; category 24 = July 2021) to capture non-linear changes throughout the Covid-19 pandemic. Due to the timing of Lifelines assessments, a few months had very few (e.g. August 2020) or no observations (e.g. February 2021). These categories were merged with the previous month (e.g. “July/August 2020”) or dropped (February 2021).

#### Time-varying moderators

Employment situation is a categorical variable comparing those “working as usual”, i.e. those whose employment situation did not change due to the pandemic (including full- and part-time work), with those “working full-time from home” (those combining working from home with working from their usual location were not included in this category, as we were interested in grasping the effects of fully adjusting to working from home), “retired”, “unemployed”, “occupationally disabled”, and “others” (including individuals on maternity leave, homemakers, students, etc.).

Alcohol consumption was assessed in 13 waves. The questions referred to the amount of alcohol consumed: “How many glasses of alcohol did you drink in the past 7 days?” or “in the past 14 days” from wave 7 onwards. In the Netherlands, standard glass is defined as containing roughly 10 g of alcohol [32]. We assessed the following categories: “Abstinence” (no alcohol consumption since last observation); “moderate drinking” (< 1.5 drinks/day on average); “heavy drinking” (1.5–3 drinks/day) [32]; and “hardcore drinking” (> 3 drinks/day) [33].

#### Time-constant confounders

Gender (male/female); *Age* at baseline, categorised in age groups (< 40, 41–50, 51–60, 61–70 and > 70); and educational level, categorised based on the Dutch educational system: “low” (up to general secondary education), “middle” (secondary vocational education, or higher general and pre-university education) or “high” (higher professional education or university education).

#### Pre-pandemic mental disorders

Based on data from wave 4 of the Lifelines Cohort Study (gathered between 2014 and 2017), gold-standard assessments of “major depressive disorder” and “generalized anxiety disorder” were combined in the variable pre-pandemic mental disorder.

#### Pre-pandemic alcohol consumption

Based on wave 5 of the Lifelines Cohort Study (gathered between 2016 and 2019), the following drinking patterns were assessed: “Abstinence”, “Moderate drinking” and “Heavy or Binge drinking” (including either “ > 1.5 glasses/day on average” or “4 or more drinks/occasion” in the last month [34]).

### Statistical analyses

Our analytical strategy was based on the following steps: first, linear regression models (OLS) accounting for fixed-effects (FE) were fitted [35], with depression and anxiety symptoms as separate outcome variables, and the lockdown period as the main independent variable. Based on these FE models, the predicted symptoms at every time point were estimated.

Second, depression and anxiety symptoms were combined in an overall “mental health score” and the potential roles of the employment situation and alcohol consumption as moderators were tested by adding two-way interactions with the main independent variable. Again, the predicted symptoms at each time point for these subgroups of individuals were estimated.

Last, fluctuations in mental health [36] were studied as sensitivity analyses. To do so, the intra-individual standard deviation of mental health scores across waves was calculated. Next, this variable was regressed on a series of covariates that included mental health scores at baseline, as well as pre-pandemic mental disorders and drinking patterns. Missing values in pre-pandemic variables (25% and 40%, respectively) were imputed using multiple imputations by chained equations (MICE) with the rest of the covariates, including the outcome to improve the model’s predictive power. However, the imputed model was estimated using only observed values of the outcome [37], hence the somewhat lower sample size (*n* = 65,503). All analyses were performed with Stata 13.

## Results

The mean baseline depression and anxiety scores of the study population, broken down by the main variables of interest, are shown in Table 1. Overall, baseline levels are relatively low, since most individuals did not report any depression or anxiety symptoms at all (67 and 70%, respectively). Yet, the dispersion is pretty large, as shown by the standard deviations, because some groups -e.g. those with occupational disabilities or pre-existing mental health issues- did report high symptom scores. Thus, meaningful differences between groups are observed: unemployed individuals reported significantly higher levels than those working as usual (whereas retired individuals reported significantly lower symptoms), and younger individuals’ depression and anxiety levels doubled, and tripled (respectively) that of the oldest group. Differences by gender and living arrangement are also relatively large, with women and those living alone reporting significantly higher depression and anxiety symptoms than their counterparts, whereas differences by educational level or pre-pandemic drinking patterns are rather small.

**Table 1:** Main variables of interest by depression and anxiety symptom score at baseline (*n* = 76,759)*

	*n *(%)	Missing (%)	Depression score (mean (SD))	Anxiety score (mean (SD))
Gender		0 (0.00%)		
Male	30,032 (39.11%)		0.46 (1.04)	0.56 (1.28)
Female	46,763 (60.89%)		0.67 (1.19)	0.86 (1.53)
Age (mean; SD)	53.66 (12.97)			
Age group		0 (0.00%)		
< 40	12,426 (16.18%)		0.81 (1.37)	1.12 (1.70)
41–50	15,890 (20.69%)		0.66 (1.18)	0.87 (1.54)
51–60	26,609 (34.65%)		0.59 (1.13)	0.72 (1.42)
61–70	13,993 (18.22%)		0.42 (0.93)	0.50 (1.19)
> 70	7877 (10.26%)		0.39 (0.89)	0.42 (1.08)
Education years (mean; SD)	12.41 (2.37)	2846 (3.71%)		
Educational attainment		2846 (3.71%)		
Low	19,235 (25.05%)		0.60 (1.17)	0.71 (1.44)
Middle	29,159 (37.97%)		0.60 (1.15)	0.77 (1.47)
High	25,555 (33.28%)		0.53 (1.05)	0.71 (1.39)
Employment Status		114 (0.15%)		
Work (as usual)	31,823 (41.44%)		0.55 (1.07)	0.75 (1.44)
Work (from home)	18,414 (23.98%)		0.55 (1.05)	0.76 (1.42)
Retired	15,269 (19.88%)		0.38 (0.87)	0.42 (1.09)
Unemployed	2,153 (2.80%)		0.97 (1.52)	1.12 (1.79)
Occupationally disabled	1945 (2.53%)		1.74 (1.98)	1.73 (2.09)
Other	7077 (9.22%)		0.88 (1.43)	1.00 (1.67)
Living arrangement		3682 (4.79%)		
With partner/family	65,379 (85.13%)		0.56 (1.09)	0.72 (1.42)
Living alone	7734 (10.07%)		0.81 (1.40)	0.84 (1.55)
Drinking pattern		13,601 (17.71%)		
Abstainer	20,615 (26.84%)		0.66 (1.21)	0.79 (1.49)
Moderate	36,605 (47.67%)		0.51 (1.04)	0.66 (1.35)
Heavy Drinking	5071 (6.60%)		0.55 (1.06)	0.72 (1.42)
Hardcore drinking	903 (1.18%)		0.72 (1.37)	0.85 (1.61)
Pre-pandemic drinking pattern		30,721 (40.00%)		
Abstainer	8974 (11.69%)		0.64 (1.21)	0.77 (1.47)
Moderate	29,072 (37.86%)		0.48 (0.99)	0.62 (1.31)
HD or BD	8028 (10.45%)		0.54 (1.09)	0.68 (1.40)
Pre-pandemic mental health		19,389 (25.25%)		
No disorder	53,626 (69.83%)		0.48 (0.97)	0.62 (1.30)
Depression/anxiety disorder	3780 (4.92%)		1.60 (1.87)	1.98 (2.13)

*T tests and *F* tests (Anova) were used to test differences between groups. They were all statistically significant

### Depression and anxiety symptoms during lockdown

An overview of the most relevant preventive measures implemented in the Netherlands during the observation period is shown in the appendix (Table A1). Table 2 shows the shifts during the lockdown period in depression symptoms (Model 1), anxiety symptoms (Model 2), and combined mental health symptoms (Model 3). Although the three outcome variables have different ranges, hence the different coefficient sizes, their temporal patterns are quite similar, with only slight differences between depression and anxiety in certain periods. For example, between September and November 2020 depression symptoms remained unchanged (*β* = 0.00; 95% CI 0.00; 0.01), whereas anxiety symptoms slightly decreased (*β* = − 0.03; 95% CI − 0.04; − 0.02).

**Table 2:** Effects of the lockdown period on depression (Model 1), anxiety (Model 2) and overall mental health symptoms (Model 3)

	Depression		Anxiety		Mental health	
	Model 1 (*n* = 841,547)		Model 2 (*n* = 841,696)		Model 3 (*n* = 841,370)	
	β	CI 95%	β	CI 95%	β	CI 95%
*Lockdown period (April 2020)*						
May 2020	− 0.04**	(− 0.04; − 0.03)	− 0.11**	(− 0.12; -0.10)	− 0.12**	(− 0.13; − 0.11)
June 2020	− 0.04**	(− 0.04; − 0.03)	− 0.11**	(− 0.12; − 0.10)	− 0.13**	(− 0.14; − 0.12)
July/August 2020	− 0.06**	(− 0.06; − 0.05)	− 0.16**	(− 0.17; − 0.15)	− 0.19**	(− 0.20; − 0.18)
September/October 2020	0.00	(− 0.01; 0.00)	− 0.03**	(− 0.04; 0.02)	− 0.03**	(− 0.04; − 0.02)
November 2020	0.00	(0.00; 0.01)	− 0.03**	(− 0.04; 0.02)	− 0.03**	(− 0.04; − 0.02)
December 2020	0.01*	(0.00; 0.01)	− 0.03**	(− 0.04; 0.02)	− 0.02**	(− 0.03; − 0.01)
January 2021	0.07**	(0.06; 0.08)	0.01	(0.00; 0.02)	0.07**	(0.05; 0.08)
March 2021	0.12**	(0.12; 0.13)	0.07**	(0.06; 0.08)	0.17**	(0.15; 0.18)
April 2021	0.06**	(0.05; 0.06)	0.00	(− 0.01; 0.01)	0.05**	(0.03; 0.06)
May 2021	0.04**	(0.04; 0.05)	− 0.02**	(− 0.03; 0.00)	0.02*	(0.00; 0.03)
June/July 2021	0.05**	(0.04; 0.06)	− 0.02**	(− 0.03; − 0.01)	0.01	(− 0.01; − 0.02)

OLS fixed-effects models

*p value < 0.05; **p value < 0.01

**Fig. 1:**
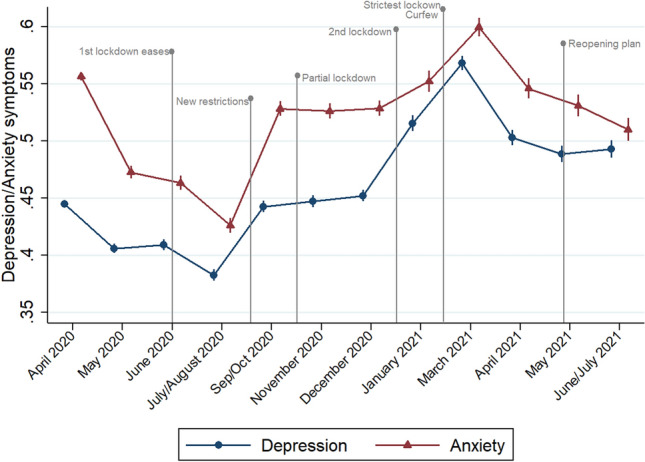
Anxiety and depression symptoms during the COVID-19 lockdown

As shown in Fig. 1, depression and particularly anxiety symptoms were relatively high at the beginning of the observation period and decreased steadily in the subsequent months, consistent with the negative coefficients shown in Table 2, with a dip in July/August 2020 (*β* = − 0.06; 95% CI − 0.06; − 0.05, and *β* = − 0.16; 95% CI − 0.17; − 0.15, respectively). However, both increased after the summer -anxiety more steeply- coinciding with a tightening of preventive measures and followed by a plateau during autumn. A third phase started in the winter of 2021, coinciding with the strictest period of the lockdown, during which a curfew was imposed and all non-essential businesses were closed: both anxiety and depression escalated and peaked by March 2021, when they surpassed the levels reported in baseline. Thus, depression symptoms increased by 30% and anxiety by 7% compared with baseline levels.

Finally, both symptom values sharply decreased afterwards, with lower anxiety and depression levels by April 2021 (anxiety even returning to baseline levels), and a subsequent steady decrease of symptoms -particularly anxiety- until summer. Notably, although the “reopening plan” or progressive relaxation of restrictions, including opening bars, allowing more guests at home, etc., did not start until the 28th of April 2021, schools had already reopened at the beginning of March. This, and the end of the winter season, coincided with a reduction in anxiety and depression levels. However, by the end of the observation period, the average depression levels remained 11% higher than at baseline (*β* = 0.05; 95% CI 0.04; 0.06 in June/July), whereas anxiety symptoms were slightly lower (*β* = − 0.02; 95% CI − 0.03; − 0.01).

### The moderating role of the employment situation

Due to the similarity in the patterns of depression and anxiety symptoms, interaction terms were only added to Model 3 using a combined mental health symptom score as the outcome. As shown in Table A2 in the appendix, the large number of significant interaction coefficients show that employment situation moderated the association between the lockdown and mental health.

**Fig. 2:**
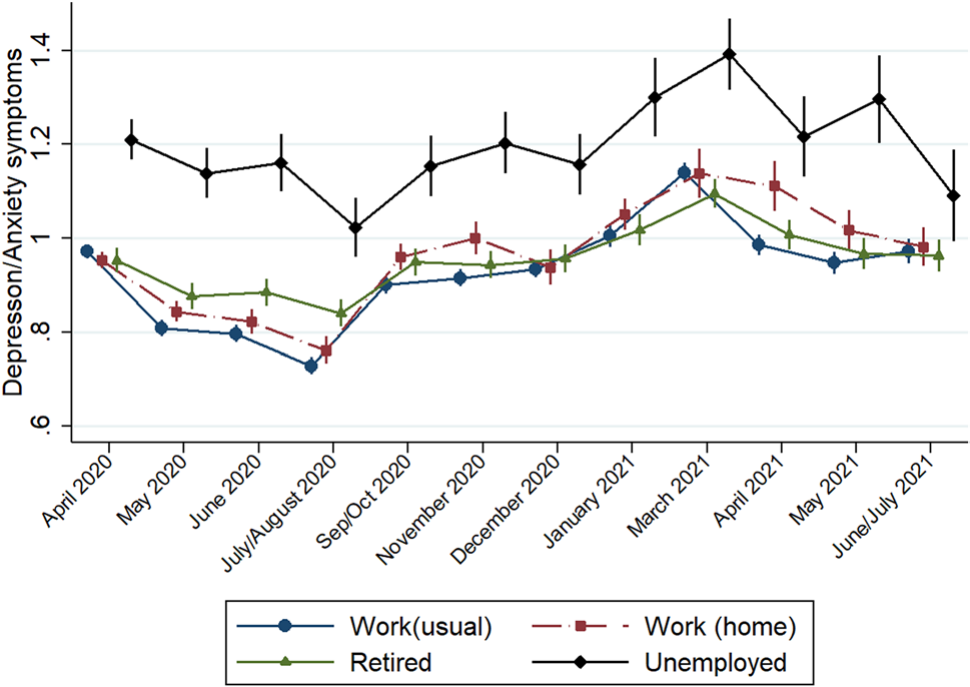
Mental health symptoms during the COVID-19 lockdown by employment situation

First, *unemployed* individuals reported a delayed improvement in their mental health after the first lockdown, in the spring of 2020: as shown in Fig. 2, their symptoms did not decrease until July/August 2020, when they abruptly did so, whereas their employed peers had started improving that May. Moreover, they showed a delayed improvement in their symptoms after the second lockdown: in May 2021, while most individuals reported decreased symptoms, this group experienced increased symptoms (*β* = 0.11; 95% CI 0.01; 0.21), and their symptoms did not decrease drastically until early summer 2021 (*β* = − 0.12; 95% CI − 0.22; − 0.01).

Second, *retired* individuals showed significant interaction coefficients in the summer of 2020 (July/August: *β* = 0.13; 95% CI 0.10; 0.16), when overall mental health improved substantially, suggesting that they experienced less improvement during the first period of relaxation of the measures. However, as Fig. 2 shows, after that period, the retired showed the most stable temporal pattern, with relatively low anxiety and depression symptoms, similar to those who were working as usual.

Third, for those working from home, mental health symptoms were particularly high in the autumn of 2020 and early spring of 2021. Moreover, an increasing gap with those working as usual was observed, combined with periods of convergence that coincide with the holiday periods (summer and Christmas). Thus, compared with the scores in May 2020 (*β* = 0.05; 95% CI 0.03:0.08), after the first lockdown, this group’s coefficients had increased by November 2020 (*β* = 0.10; 95% CI 0.06:0.14) and were even greater in April 2021 (*β* = 0.14; 95% CI 0.09:0.20), at the end of the strictest lockdown period, indicating an accumulation of mental health symptoms over time. Sensitivity analyses (not shown) showed that the association between lockdowns and mental health did not depend on whether these individuals lived with family or on their own.

Last, individuals with *occupationally disability* not only showed much higher levels of depression and anxiety symptoms than the others but also showed a significantly stronger deterioration of their mental health after the summer of 2020: in September/October 2020, coinciding with the tightening of preventive measures, their mental health symptoms increased by 11% compared to baseline (*β* = 0.15; 95% CI 0.07; 0.23) measures (as shown in Figure. A1). By the Spring of 2021, their depression and anxiety symptoms had increased by 28% (*β* = 0.35; 95% CI 0.25; 0.46), whereas other groups—e.g. those working as usual—showed a slight improvement (*β* = − 0.02; 95% CI − 0.05; 0.00). Interestingly, those with pre-existing mental disorders followed a similar pattern, although with somewhat higher symptom scores, suggesting that pre-existing mental disorders and occupational disability are strongly associated.

Overall, results suggest that unemployed individuals and those with occupational disabilities required some time after the relaxation of the measures in late spring 2020 to experience mental health improvements. Moreover, the latter were particularly vulnerable to the uncertainty that the pandemic sparked, judging by their strong fluctuations in mental health symptoms. Conversely, retired individuals seemed to overcome the initial shock quite fast and became less affected by the lockdown measures and the evolution of the pandemic over time. Finally, for those working from home mental health symptoms seemed to accumulate: while their mental health improved during holiday periods, their symptoms worsened coinciding with every new set of restrictions.

### The moderating role of alcohol consumption

**Fig. 3:**
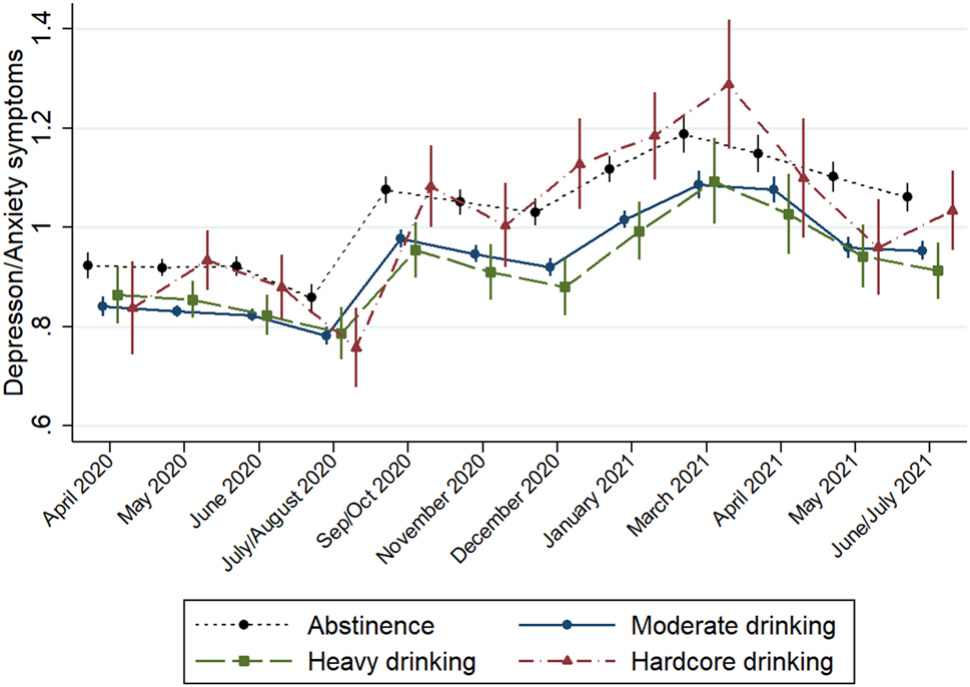
Mental health symptoms during the COVID-19 lockdown by alcohol consumption

As shown in Table A3, while the pattern did not differ significantly between those reporting *moderate* and *heavy* drinking, those drinking more than 3 drinks/day reported significantly higher instability in their mental health throughout the observation period. Figure 3 shows that individuals reporting “hardcore drinking” moved from having average symptoms during periods with more relaxed measures, such as July/August 2020 and May 2021, to report the highest anxiety and depression levels during the strictest lockdown periods (by March 2021, mental health symptoms had increased by 44% compared to baseline levels). In some cases, these changes were very drastic, e.g. between July and September 2020, they increased steeply, but they decreased rapidly between March and May 2021. In contrast, abstainers reported relatively high levels of depression and anxiety but a more stable pattern overall, including during periods of relaxed measures (e.g. the summer of 2020 and spring of 2021).

Altogether, our moderation analyses show clear differences by employment situation and, to a lesser degree, by drinking pattern in the association between the lockdowns and mental health. Furthermore, symptom fluctuation throughout the observation period was relevant, as this indicates higher mental instability, and, therefore, higher vulnerability, for some groups. Hence, sensitivity analyses were conducted to identify the main determinants of mental health fluctuation during lockdowns.

### Fluctuations in mental health during lockdown periods

As shown in Table A4 in the appendix, pre-existing mental disorders and mental health at baseline were the strongest determinant of mental health fluctuations, i.e. stronger fluctuations coincided with higher depression and anxiety symptoms before and at the beginning of the pandemic. Furthermore, fluctuations were particularly large among unemployed individuals and, to a lesser degree, those working from home, while retired individuals reported lower levels of fluctuation. Interestingly, the association between disability and mental health fluctuations weakened after pre-existing mental disorders were accounted for, suggesting that occupational disability was partly due to mental health conditions.

Meanwhile, alcohol consumption showed a gradient in the association with fluctuations in mental health: while abstainers did not differ substantially from those drinking moderately, the results show that those who reported heavy, and, particularly, hardcore drinking experienced significantly stronger mental health fluctuations, even after accounting for pre-pandemic drinking patterns and other covariates, suggesting that alcohol consumption independently contributed to mental health instability.

## Discussion

Our study, which relies on a very large sample and a robust longitudinal design provides detailed analyses of the trends in depression and anxiety symptoms that people experienced during lockdown periods. Moreover, by focusing on individuals’ working situations during lockdowns, as well as their mental health and drinking patterns before and during the pandemic, this study provides a thorough analysis of the main determinants of depression and anxiety in the context of the pandemic. Our results show that depression and anxiety symptoms fluctuated throughout lockdown periods, with relatively low values during summer periods, when restrictions eased, and a peak during the winter of 2021, coinciding with the strictest lockdown period in the Netherlands. Although these fluctuations were relatively small for the population as a whole, they particularly differed by employment situation and alcohol consumption, suggesting that various subgroups reacted to the pandemic and lockdowns differently.

First, our results showed increases in overall levels of depression and anxiety symptoms during the first lockdown in the Netherlands, and an overall improvement in mental health over the subsequent few months. This aligns with studies showing an initial shock, allegedly due to the fear and uncertainty that the outbreak of Covid-19 entailed [1, 2, 11], followed by an improvement in mental health [5, 6]. However, these early studies failed to capture the subsequent rapid increase in symptoms after the first pandemic summer, and the peak in depression and anxiety symptoms between January and March 2021, during the strictest lockdown period, as more recent studies showed [28, 38, 39]. Our findings align with the latter in suggesting that the measures implemented during that period -a curfew from 9 PM to 5 AM, only one visitor allowed at home, and the closing of all public buildings and non-essential shops- were strongly associated with the deterioration of mental health of the observed population [38]. In addition, we observed an overall rapid improvement during the spring of 2021. Although restrictions were not entirely lifted until the end of May, a “cautious easing” of the lockdown started at the beginning of March, with schools reopening and shops and contact-based professionals permitted to receive customers by appointment. This, alongside the first vaccinations, may have sparked hope for a return to normal life that permitted an improvement of mental health symptoms. Alternatively, it could be argued that mental health symptoms tend to decrease after every winter [40, 41]. However, our results show a clear deterioration of mental health, particularly regarding the prevalence of depression symptoms, in the spring of 2021 compared with that of 2020, suggesting that the length of the lockdown was relevant [28].

Second, how strongly mental health fluctuated during the pandemic varied by individuals’ employment situation. In line with theories on perceived control [7], the fluctuations were particularly strong among unemployed individuals, who experienced a stronger deterioration in mental health during lockdowns as well as a slower improvement during periods of relaxed restrictions. Similarly, individuals who were working from home not only experienced a stronger deterioration of their mental health, but their mental health symptoms accumulated with every new set of restrictions. In turn, those with occupational disabilities had high baseline levels of depression and anxiety that worsened during lockdowns. Interestingly, the differences in reaction to lockdowns between unemployed and retired individuals—who, after an initial shock, showed a more resilient pattern during the winter of 2021—support the hypothesis that the deterioration of mental health during the pandemic was related to uncertainty and lack of control [7, 8], which often characterizes unemployment [42]. In contrast, the relatively generous retirement pension system in the Netherlands [43] may have buffered the impact for retired individuals.

Third, results show that those reporting “hardcore drinking” suffered higher mental health instability, and that it was strongly related to the lockdowns, supporting the hypothesis that alcohol abuse increases the risk for depression [19, 21], and mood instability [20]. Abstainers were consistently characterized by “relatively stable distress”, i.e. more symptoms but smaller fluctuations than those drinking moderately. We did not observe different patterns for those with moderate and heavy drinking patterns, suggesting that the threshold of 1.5 drinks per day was insufficient to identify a hazardous drinking pattern, at least in terms of mental health in our study population. However, “hardcore drinking” offered a better indicator, probably because it is a proxy for alcohol intoxication, which has been shown to entail higher risks (and present a steeper socioeconomic gradient) than mild, frequent abuse [44, 45].

Finally, sensitivity analyses showed that individuals with pre-existing mental disorders experienced more fluctuations in their mental health. Although fluctuation could also indicate recovery, these individuals reported more mental health symptoms during the observation period, which seems to contradict the idea of better adjustment among those with pre-existing mental disorders [16]. Instead, our findings suggest that mental health symptoms tended to accumulate with every new lockdown, as previous studies reported [46], and in line with the concept of “accumulation of risks” [47].

This study has several limitations. First, although the depression and anxiety symptoms were based on DSM-V criteria, the duration of the symptoms assessed in the Lifelines Covid-19 Questionnaire did not match the standard definition of major depressive or generalised anxiety disorders, as we were interested in short-term responses to the pandemic. This disallowed straight comparisons of pre- and post-pandemic levels and therefore hampered the interpretability of mental health during the pandemic. Second, our alcohol assessment relied on a self-reported average of glasses consumed since the last observation but did not assess the intensity of drinking, i.e. acute intoxication. However, by establishing different cut-off points, we were able to identify a subgroup with a particularly hazardous drinking pattern. Third, despite our longitudinal design, a direct causal effect of the lockdowns on mental health cannot be inferred, as the associations could be confounded by unobserved variables, such as personality traits or genetic predispositions (e.g. individuals with different chronotypes could adjust differently to work-related transformations, such as working from home [39]). Last, due to data availability, we could not assess some potentially relevant variables, such as financial stress or decreased social life, which may have been strongly associated with mental health during the pandemic. Further research should focus on longitudinal designs to disentangle the numerous factors associated with mental health in the context of the Covid-19 pandemic.

## Conclusions

This longitudinal study shows that depression and anxiety symptoms strongly increased in the Netherlands during the COVID-19 pandemic, particularly during the strictest lockdown period, in the winter of 2021. However, the deterioration of mental health during the pandemic was not homogeneous across the population, but particularly strong among unemployed individuals, as well as among those with risky alcohol consumption patterns and pre-existing mental disorders. An accurate profiling of subgroups at higher risk for higher depression and anxiety, as well as stronger mood fluctuations, is key to designing tailored preventive policies in exceptional contexts like the Covid-19 lockdown to prevent further deterioration of mental health in similar scenarios in the future.

## Supplementary Information

Below is the link to the electronic supplementary material.

Supplementary file1 (PDF 405 KB)

## Data Availability

The datasets generated and analysed during the current study were provided by Lifelines under licence. Access to the data can be granted under licence by Lifelines and the authors will share the codes used to produce the results presented in this paper upon request.
