# Supplementary material for: Changes in anxiety and depression symptoms during the Covid-19 lockdown in the Netherlands. The moderating role of pre-existing mental health, employment situation and alcohol consumption
Source: Soc Psychiatry Psychiatr Epidemiol. 2023 Apr 6;58(10):1561–71. doi: 10.1007/s00127-023-02480-6 (PMC10079151; doi:10.1007/s00127-023-02480-6)
Supplement: Supplementary file 1 — Supplementary file1 (PDF 405 KB) [file 127_2023_2480_MOESM1_ESM.pdf]

Appendix (online resources)

Table A1. Timeline of preventive measures in the Netherlands (March 2020 – July 2021)

Date	Event
27/02/2020	First positive cases of Covid-19 in the Netherlands
06/03/2020	First death due to Covid-19 in the Netherlands
09/03/2020	Government announced a ban on hand-shaking<br>People in Noord -Brabant advised to work from home for the next seven days
10/03/2020	Government changes its travel advice to Italy to orange (essential travels only)
12/03/2020	Work from home guideline extended to the whole country<br>Gatherings of 100 people were banned (extended later)
15/03/2020	**“Intelligent lockdown” in the Netherlands began** (extended later)<br>Schools closed<br>Cafes, restaurants, sports and sex clubs were asked to close<br>People instructed to keep 1.5 meters distance from each other
16/03/2020	Funerals limited to 30 mourners<br>Package of measures to support workers and businesses affected by the pandemic.
17/03/2020	All countries were changed to code orange (essential journeys only)<br>EU member states agree to close external border for 30 days
18/03/2020	Education minister cancelled end-of-year exams for children in the last year of primary school
19/03/2020	Visits to nursing homes banned
23/03/2020	**Stricter lockdown rules announced** (extended later)<br>Groups of more than three people in public are banned<br>Bans on mass gatherings extended<br>Shops introduced measures to keep people 1.5 meters apart<br>Contact professions ordered to close
24/03/2020	Summer exams for school leavers cancelled
25/03/2020	Closure of university and colleges extended
31/03/2020	**Lockdown measures extended**
21/04/2020	**Lockdown measures extended**<br>Bans on mass gatherings / public events extended
29/04/2020	Some groups of are allowed to play sports again
06/05/2020	The cabinet publishes its plan for a **staged relaxation of the 'intelligent lockdown'**
11/05/2020	Most contact professions reopened again<br>Primary schools, childcare providers reopened<br>Some hairdressers, libraries reopened again
19/05/2020	Lockdown measures extended
01/06/2020	**Relaxation of many measures, easing of lockdown**<br>People may meet outside with 1.5 meters distance apart<br>Buildings that are open to the public can admit up to 30 people at a time<br>**Restaurants, bars and cafes open under several conditions** (max 30 guests, reservations, 1.5-meter distance)

	Cinemas, theatres and concert halls can open under several conditions (similar to above)
	Passengers aged 13 and over required to wear face mask on public transport
02/06/2020	Secondary schools reopened again
03/06/2020	Self-quarantine at home required for two weeks when coming back from outside Europe
15/06/2020	Secondary schools (MBO & HBO) and universities reopened to a limited extent
01/07/2020	**Moving from an intelligent lockdown to less restrictive rules**
	Up to 100 people may be in the same space indoors (more if everyone reserves)
	Up to 250 people may be in the same outdoors space
16/07/2020	Ease of travel ban on the entry of partners from third countries engaged in long-distance relationship with someone with the Dutch nationality or EU-citizen who live in The Netherlands
06/08/2020	Restaurants, cafés and bars must use reservations, pre-entry health checks and assigned seating
18/08/2020	**Government announced tightening up of measures**
	Limiting to a maximum of 6 guests at home
	Emphasized on advice to work from home whenever possible
	Self-quarantine period reduced from 14 days to 10 days (countries with orange travel advisory)
01/09/2020	Ban on group singing, chanting and shouting
28/09/2020	Limiting to a maximum of 3 guests at home. The number of people in 1 room is limited to 30 people.
	Sport clubs closed
01/10/2020	Government provided support and recovery package for businesses and employees
14/10/2020	**Partial lockdown enacted**
	Indoors and outdoors a group must have no more than 4 people from different households
	**All establishments that serve food and drinks must close (except for combined function locations)**
	**Ban on alcohol sale, delivery and consumption in public spaces between 20:00 and 7:00**
	Events are banned (with exceptions of food markets, trade fairs, conferences, cinemas and theatres, etc.)
03/11/2020	Temporary tightening of partial lockdown (extended later)
	Limiting to a maximum of 2 guests at home
	All venues that are normally open to the public are closed (e.g. museums, theatres, etc.)
	People aged 18 and older may only take part in sports individually or in groups of 2
17/11/2020	**Partial lockdown extended, but slightly relaxed**
	Museums, theatres, swimming pools and amusement parks reopen
	Limit of maximum guests indoors brought back to 3
	People aged 18 and older may only take part in sports individually or in groups of 4
08/12/2020	Partial lockdown prolonged, continued during Christmas and New Years
20/12/2020	Flights carrying passengers from the U.K. banned due to new strain
08/01/2021	**First Covid-19 vaccination started**
12/01/2021	**Partial lockdown extended**
20/01/2021	**Lockdown measures tightened in response to concerns about new variants of virus**
	Strict travel advisory: “Do not travel abroad and do not book any trips abroad until 31st March 2021”
23/01/2021	**Curfew enforced from 20:30 to 04:30**
	Government announced flight bans to many countries (e.g. U.K., South Africa, Dominican Republic)
02/02/2021	**Extension of lockdown.** Limit of maximum guests indoors brought back to 1
08/02/2021	**Curfew extended.** Primary schools, childcare providers reopened fully
10/02/2021	Non-essential shops allowed to offer their customers the option to collect their order in person
23/02/2021	**Cautious easing of lockdown. Curfew extended**

01/03/2021	Secondary schools and institutions of secondary vocational education (MBO) reopened
03/03/2021	Contact-based professions reopened, with restrictions. Shops can receive customers by appointment
08/03/2021	Lockdown, curfew, and all other current coronavirus measures are extended
23/03/2021	Lockdown (as well as all other current coronavirus measures) further extended
28/04/2021	**First step of the reopening plan: curfew is lifted**
	Maximum limit of indoor visitors back to 2 (from 1)
	Outdoor seating areas at cafes and restaurants partially reopen
	Shops and market reopen (appointment mandatory)
	Students in higher education are allowed to go to in-person classes once a week
15/05/2021	Travelling to countries with a low rate of infection is now allowed
19/05/2021	**Second step of the reopening plan**
	Reopening of indoor sports facilities
	Outdoor sports and leisure locations are allowed again
	**Reopening of all contact-based industries**
05/06/2021	**Third step of the reopening plan**
	**Alcohol ban reduced to between 22.00 and 6.00 (from 20.00 - 6.00)**
	Maximum limit of indoor visitors back to 4 (from 2). Cultural venues are allowed to reopen again
26/06/2021	**Fourth step of reopening plan**
	All establishments are allowed to reopen again (with 1.5-meter distance rule)
	**Restrictions on number of visitors at home and group size are lifted**
	Face mask requirement dropped in most situations
10/07/2021	**Reopening plan is cancelled** due to a sharp rise in COVID-19 cases
	All restaurants and bars must close from midnight until 6.00, and discos and nightclubs close again

Table A2. Effects of the lockdown period on mental health symptoms. Main effects (Model 0) and interaction with employment situation (Model 1). OLS fixed-effects models.

	Model 0 (n=841,370)		Model 1 (n=841,238)	
	β	CI 95%	β	CI 95%
Lockdown Period (April 2020)				
May 2020	-0.12**	(-0.13; -0.11)	-0.16**	(-0.18; -0.15)
June 2020	-0.13**	(-0.14; -0.12)	-0.17**	(-0.19; -0.16)
July/August 2020	-0.19**	(-0.20; -0.18)	-0.24**	(-0.26; -0.22)
September/October 2020	-0.03**	(-0.04; -0.02)	-0.07**	(-0.09; -0.05)
November 2020	-0.03**	(-0.04; -0.02)	-0.06**	(-0.07; -0.04)
December 2020	-0.02**	(-0.03; -0.01)	-0.04**	(-0.06; -0.02)
January 2021	0.07**	(0.05; 0.08)	0.03**	(0.01; 0.06)
March 2021	0.17**	(0.15; 0.18)	0.17**	(0.15; 0.19)
April 2021	0.05**	(0.03; 0.06)	0.01	(-0.01; 0.03)
May 2021	0.02*	(0.00; 0.03)	-0.02*	(-0.05; 0.00)
June/July 2021	0.00	(-0.01; -0.02)	0.00	(-0.02; 0.03)
Employment situation (Employed)				
Work from home			-0.02*	(-0.04; 0.00)
Retired			-0.02	(-0.06; 0.02)
Unemployed			0.24**	(0.19; 0.28)
Disabled			0.93**	(0.88; 0.97)
Lockdown Period ## Employment				
May 2020##Work from home			0.05**	(0.03; 0.08)
June 2020##Work from home			0.04**	(0.01; 0.07)
July/August 2020##Work from home			0.05*	(0.02; 0.08)
September/October 2020##Work from home			0.08**	(0.04; 0.11)
November 2020##Work from home			0.10**	(0.06; 0.14)
December 2020##Work from home			0.02	(-0.02; 0.06)
January 2021##Work from home			0.06**	(0.02; 0.11)
March 2021##Work from home			0.02	(-0.04; 0.07)
April 2021##Work from home			0.14**	(0.09; 0.20)
May 2021##Work from home			0.09**	(0.04; 0.14)
June/July 2021##Work from home			0.03	(-0.02; 0.08)
May 2020##Retired			0.09**	(0.06; 0.11)
June 2020##Retired			0.11**	(0.08; 0.13)
July/August 2020##Retired			0.13**	(0.10; 0.16)
September/October 2020##Retired			0.07**	(0.04; 0.10)
November 2020##Retired			0.05**	(0.02; 0.07)
December 2020##Retired			0.04**	(0.01; 0.07)
January 2021##Retired			0.03	(0.00; 0.07)
March 2021##Retired			-0.02	(-0.06; 0.01)
April 2021##Retired			0.04*	(0.01; 0.07)
May 2021##Retired			0.04*	(0.00; 0.07)
June/July 2021##Retired			0.01	(-0.03; 0.05)
May 2020##Unemployed			0.09**	(0.03; 0.15)
June 2020##Unemployed			0.13**	(0.06; 0.19)
July/August 2020##Unemployed			0.06	(-0.01; 0.12)
September/October 2020##Unemployed			0.02	(-0.05; 0.09)

November 2020##Unemployed	0.05	(-0.02; 0.12)
December 2020##Unemployed	-0.01	(-0.08; 0.06)
January 2021##Unemployed	0.06	(-0.03; 0.15)
March 2021##Unemployed	0.01	(-0.07; 0.10)
April 2021##Unemployed	-0.01	(-0.10; 0.08)
May 2021##Unemployed	0.11*	(0.01; 0.21)
June/July 2021##Unemployed	-0.12*	(-0.22; -0.01)
May 2020##Disabled	-0.09**	(-0.16; -0.03)
June 2020##Disabled	0.02	(-0.06; 0.09)
July/August 2020##Disabled	-0.04	(-0.12; 0.04)
September/October 2020##Disabled	0.15**	(0.07; 0.23)
November 2020##Disabled	0.00	(-0.08; 0.08)
December 2020##Disabled	-0.01	(-0.01; 0.07)
January 2021##Disabled	0.06	(-0.04; 0.17)
March 2021##Disabled	0.09	(0.00; 0.18)
April 2021##Disabled	0.18**	(0.08; 0.29)
May 2021##Disabled	0.35**	(0.25; 0.46)
June/July 2021##Disabled	-0.01	(-0.12; 0.10)

* p-value < 0.05; ** p-value < 0.01

Figure A1. Mental health symptoms by employment situation and previous mental disorder

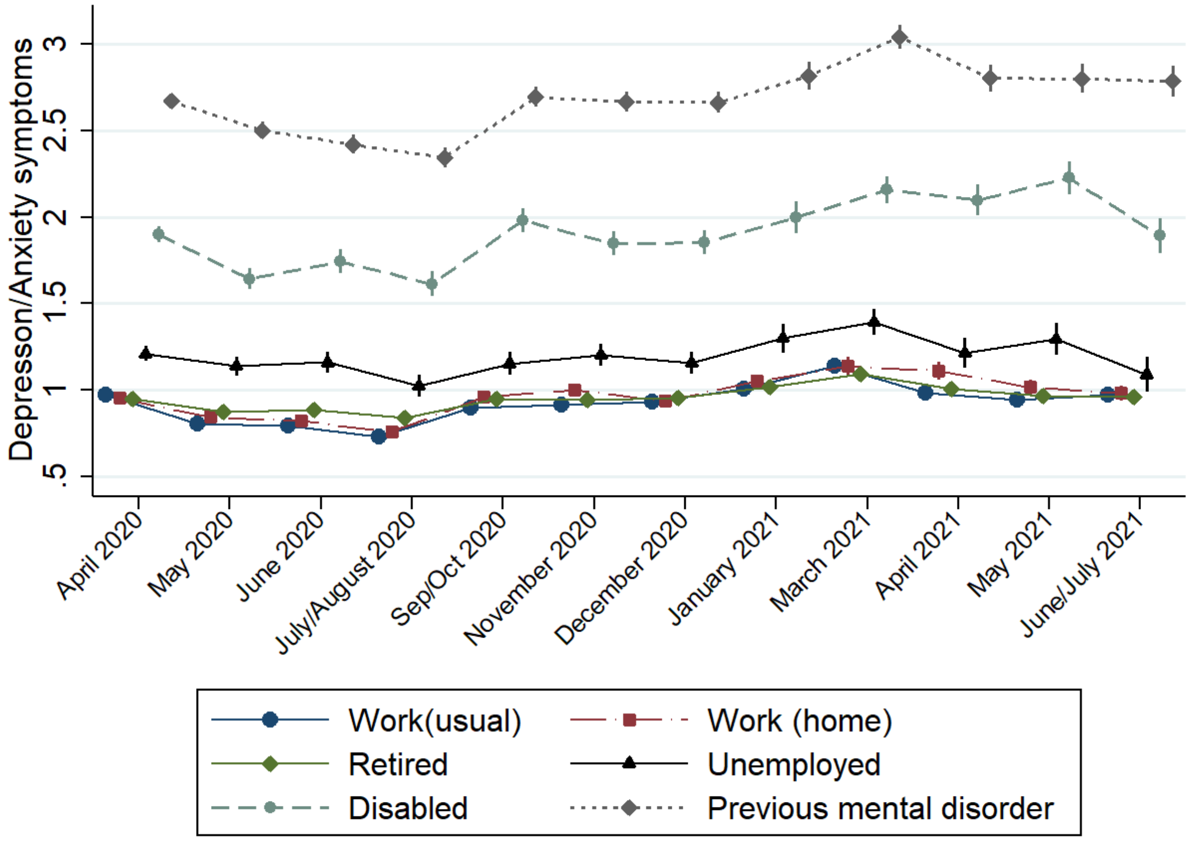

Table A3. Effects of the lockdown period on mental health symptoms. Main effects (Model 0) and interaction with alcohol consumption (Model 2). OLS fixed-effects models.

	Model 0 (n=841,370)		Model 2 (n=449,691)	
	β	CI 95%	β	CI 95%
Lockdown Period (April 2020)				
May 2020	-0.12**	(-0.13; -0.11)	-0.01	(-0.03; 0.01)
June 2020	-0.13**	(-0.14; -0.12)	-0.02	(-0.04; 0.00)
July/August 2020	-0.19**	(-0.20; -0.18)	-0.06**	(-0.08; -0.03)
September/October 2020	-0.03**	(-0.04; -0.02)	0.14**	(0.11; 0.16)
November 2020	-0.03**	(-0.04; -0.02)	0.11**	(0.08; 0.13)
December 2020	-0.02**	(-0.03; -0.01)	0.08**	(0.05; 0.10)
January 2021	0.07**	(0.05; 0.08)	0.17**	(0.15; 0.20)
March 2021	0.17**	(0.15; 0.18)	0.25**	(0.21; 0.28)
April 2021	0.05**	(0.03; 0.06)	0.23**	(0.20; 0.27)
May 2021	0.02*	(0.00; 0.03)	0.12**	(0.09; 0.15)
June/July 2021	0.00	(-0.01; -0.02)	0.11**	(0.08; 0.14)
Alcohol consumption (Moderate)				
Abstainer			0.08**	(0.05; 0.12)
Heavy drinking			0.02	(-0.04; 0.08)
Hardcore drinking			0.00	(-0.10; 0.09)
Lockdown period ## Alcohol				
May 2020##Abstainer			0.01	(-0.03; 0.04)
June 2020##Abstainer			0.02	(-0.02; 0.05)
July/August 2020##Abstainer			0.00	(-0.05; 0.04)
September/October 2020##Abstainer			0.02	(-0.03; 0.06)
November 2020##Abstainer			0.02	(-0.02; 0.06)
December 2020##Abstainer			0.03	(-0.01; 0.07)
January 2021##Abstainer			0.02	(-0.02; 0.06)
March 2021##Abstainer			0.02	(-0.04; 0.07)
April 2021##Abstainer			-0.01	(-0.06; 0.04)
May 2021##Abstainer			0.06*	(0.01; 0.11)
June/July 2021##Abstainer			0.02	(-0.02; 0.07)
May 2020##Heavy			0.00	(-0.07; 0.07)
June 2020##Heavy			-0.02	(-0.09; 0.05)
July/August 2020##Heavy			-0.02	(-0.10; 0.06)
September/October 2020##Heavy			-0.05	(-0.13; 0.03)
November 2020##Heavy			-0.06	(-0.14; 0.02)
December 2020##Heavy			-0.06	(-0.14; 0.02)
January 2021##Heavy			-0.05	(-0.13; 0.04)
March 2021##Heavy			-0.02	(-0.12; 0.09)
April 2021##Heavy			-0.07	(-0.17; 0.03)
May 2021##Heavy			-0.04	(-0.13; 0.05)
June/July 2021##Heavy			-0.06	(-0.15; 0.02)
May 2020##Hardcore			0.11*	(0.00; 0.21)
June 2020##Hardcore			0.06	(-0.05; 0.17)
July/August 2020##Hardcore			-0.02	(-0.14; 0.10)
September/October 2020##Hardcore			0.11	(-0.01; 0.23)
November 2020##Hardcore			0.06	(-0.06; 0.18)

December 2020##Hardcore	0.21**	(0.08; 0.34)
January 2021##Hardcore	0.17**	(0.05; 0.30)
March 2021##Hardcore	0.20*	(0.05; 0.36)
April 2021##Hardcore	0.03	(-0.12; 0.18)
May 2021##Hardcore	0.00	(-0.13; 0.14)
June/July 2021##Hardcore	0.08	(-0.04; 0.21)

* p-value < 0.05; ** p-value < 0.01

Table A4. Determinants of fluctuations in mental health during the observation period. OLS regression models with observed (Model 1) and imputed values[a] (Model 2).

	Model 1 (*n*=26,436)		Model 2 (*n*=49,571)	
	β	95% CI	β	95% CI
Mental health at baseline (mean)	0.24**	(0.24; 0.25)	0.25**	(0.25; 0.25)
Gender (Male)				
Female	0.18**	(0.16; 0.20)	0.16**	(0.15; 0.18)
Age (<40)				
41-50	-0.06**	(-0.09; -0.02)	-0.04**	(-0.06; -0.01)
51-60	-0.12**	(-0.15; -0.09)	-0.08**	(-0.10; -0.06)
61-70	-0.19**	(-0.23; -0.15)	-0.14**	(-0.17; -0.11)
>70	-0.18**	(-0.23; -0.12)	-0.14**	(-0.17; -0.10)
Educational Attainment (High)				
Middle	0.00	(-0.02; 0.02)	-0.01	(-0.03; 0.00)
Low	-0.01	(-0.04; 0.01)	-0.02*	(-0.04; 0.00)
Employment status (Work as usual)				
Work (from home)	0.04**	(0.02; 0.07)	0.04**	(0.02; 0.05)
Retired	-0.04*	(-0.08; -0.01)	-0.03*	(-0.06; 0.00)
Unemployed	0.06*	(0.01; 0.11)	0.09*	(0.05; 0.13)
Occupationally Disabled	0.03	(-0.03; 0.09)	0.05*	(0.00; 0.09)
Laid off due to COVID (No)				
Yes	0.08**	(0.03; 0.13)	0.08**	(0.04; 0.11)
Alcohol consumption at baseline (moderate)				
Abstainer	0.01	(-0.01; 0.03)	-0.01	(-0.03; 0.01)
Heavy drinking (1.5-3 glasses/day)	0.04*	(0.01; 0.08)	0.04**	(0.01; 0.07)
Hardcore drinking (>3 glasses/day)	0.08**	(0.00; 0.16)	0.10**	(0.03; 0.16)
Living arrangement (partner/family)				
Lives alone	0.03*	(0.00; 0.06)	0.03*	(0.01; 0.05)
Pre-pandemic mental health (no disorder)				
Depression/Anxiety disorder	0.31**	(0.27; 0.35)	0.22**	(0.19; 0.26)
Pre-pandemic alcohol consumption (moderate)				
Abstinence	-0.03*	(-0.06; -0.01)	-0.02	(-0.05; 0.00)
HD or BD	0.03*	(0.01; 0.06)	0.02	(-0.01; 0.04)

[a] Missing values imputed by means of Multiple Imputation Chained Equations (MICE).
* p-value < 0.05; ** p-value < 0.01
